# Ectopic colloid goiter in mediastinum with normal thyroid gland

**DOI:** 10.1186/s13019-024-02574-5

**Published:** 2024-02-20

**Authors:** Jagannath Kolwalkar, Dattaprasad Samant, Shirish Borkar, M. Sunil Chandra Vidyasagar, Jagadeesh N. Vaggar

**Affiliations:** Department of General and Cardiothoracic Surgery, Goa Medical College, Goa, India

**Keywords:** Ectopic thyroid, Mediastinum, Colloid goiter

## Abstract

Ectopic thyroid tissue is a rare developmental abnormality involving aberrant embryogenesis of the thyroid gland during passage from the primitive foregut to the pretracheal position. The most frequent position is the base of the tongue (lingual thyroid); however, it has been described in other sites, such as the submandibular region, trachea, mediastinum, and subdiaphragmatic regions.

Here, we report a case of an adenomatous goiter that developed in mediastinal thyroid tissue without any connection to the pretracheal thyroid gland.

## Introduction

Ectopic thyroid tissue is a rare clinical entity. The incidence is less than 1% of mediastinal tumors [1].

Ectopic thyroid tissue can coexist with eutopic thyroid, even if the majority of cases occur without cervical location of thyroid. Less than 15 cases have been reported in last 5 decades [1].

Finding goitrous mediastinal ectopic tissue with an orthotopic thyroid gland is even rarer [2].

Masses in the anterior mediastinum are usually thymoma, lymphoma, germ cell tumors [2].

Thus, we want to highlight the importance of considering ectopic colloid goiter as one of the differential diagnosis of anterior mediastinal mass even though its rare.

## Case report

A 63-year-old male underwent a HRCT (High-resolution computed tomography) thorax examination when he had high fever and persistent cough. Incidentally detected was a mediastinal mass.

The patient was asymptomatic.

Physical examination was unremarkable, and the thyroid gland was not clinically palpable. Laboratory tests showed no abnormalities. CT scan revealed an 8.4 × 7.1x6.6 cm mass with central necrosis and peripheral calcifications in the anterior mediastinum. Posteriorly, the mass abutted the aortic arch and origins of the right brachiocephalic trunk, left subclavian and common carotid arteries, and the mass displaced the anterior wall of the trachea.

CT-guided Trucut biopsy showed closely packed thyroid follicles filled with colloid. The thyroid function test was normal, TSH level was 1.6mIU/L.

The patient underwent excision of the mass via midline sternotomy. A large mass 10 × 8 cm well circumscribed in the anterior mediastinum was carefully dissected from the common carotid artery and left innominate vein and trachea.

There was no connection to the normally situated thyroid gland in the neck, which was grossly normal in size with no enlargement or nodularity.

Postoperative was uneventful.

**Fig. 1:**
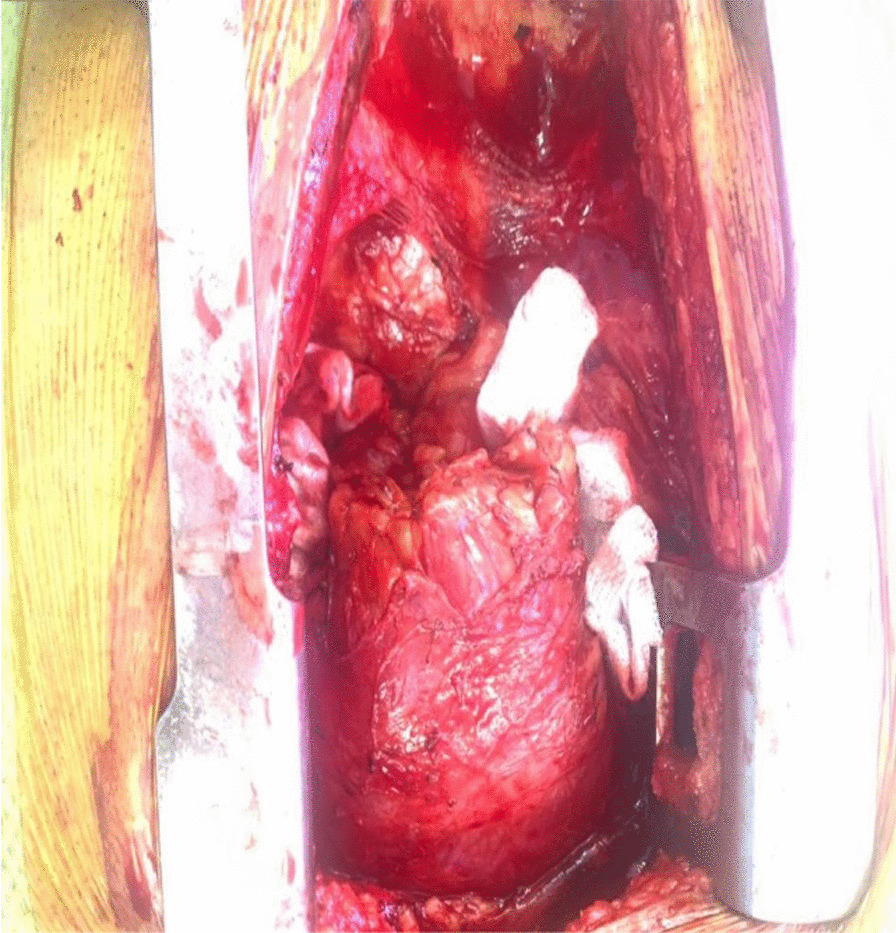
(Intra-op picture showing large anterior mediastinal mass abutting the arch of aorta)

**Fig. 2:**
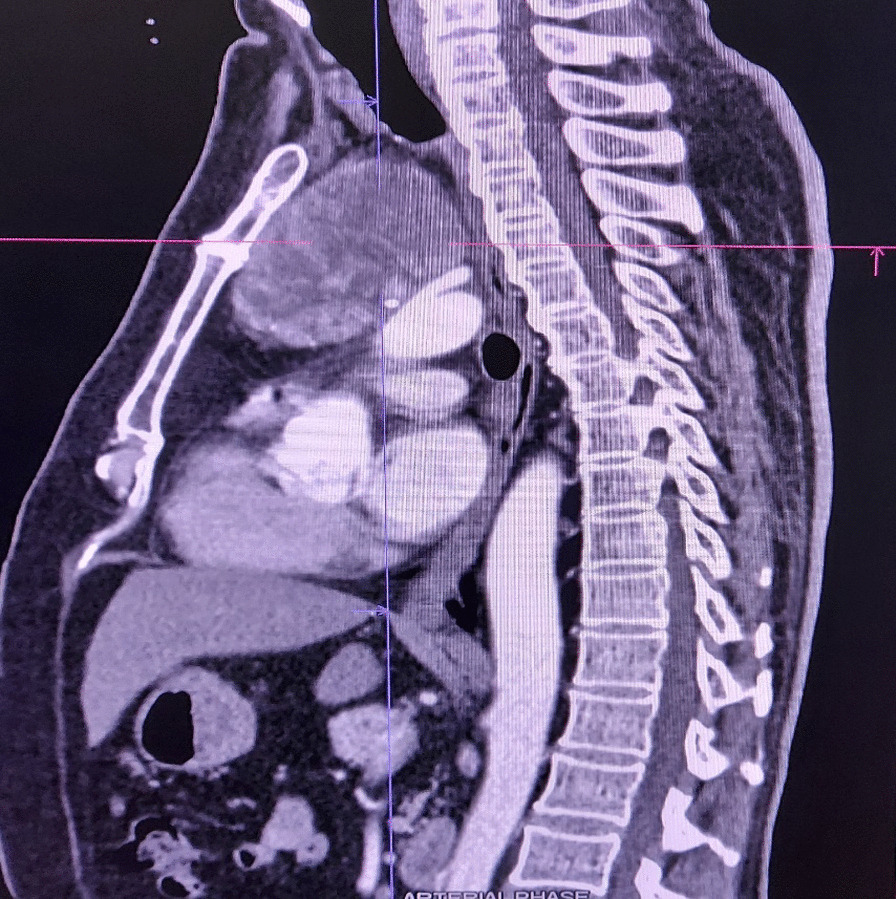
(CT scan revealing a mass with central necrosis and peripheral calcifications with normal looking thyroid gland)

**Fig. 3:**
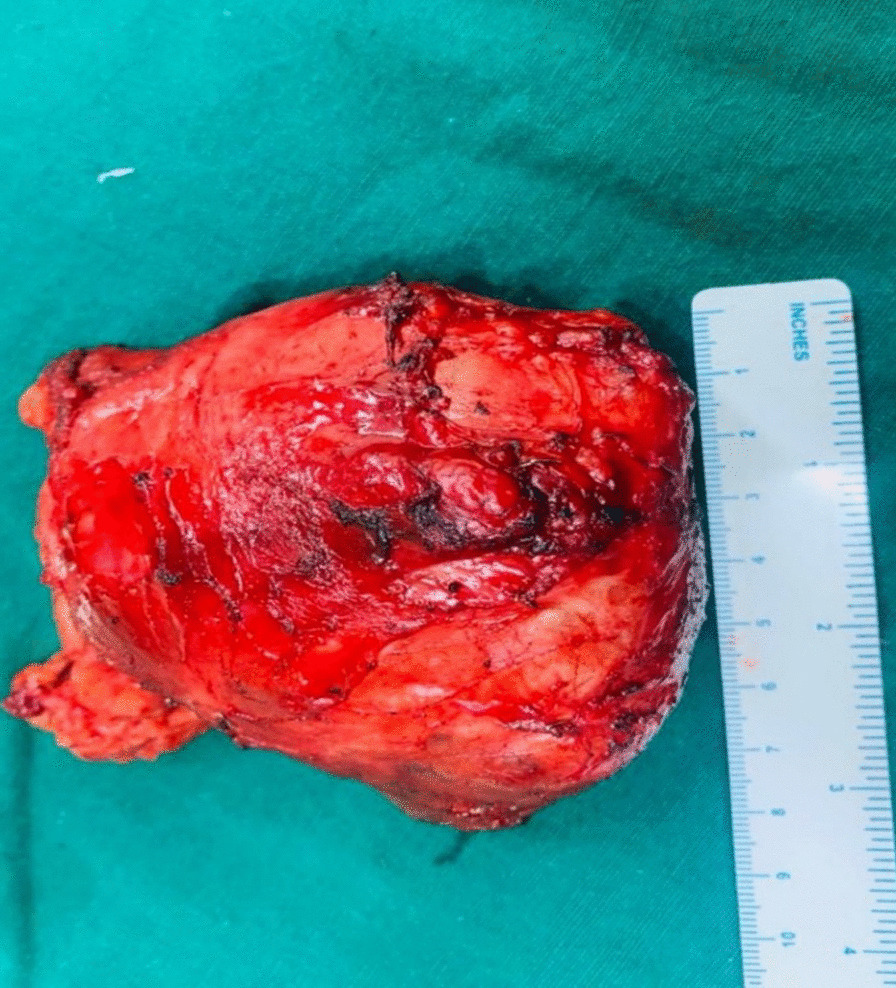
(10 × 8 cm mass carefully excised)

**Fig. 4:**
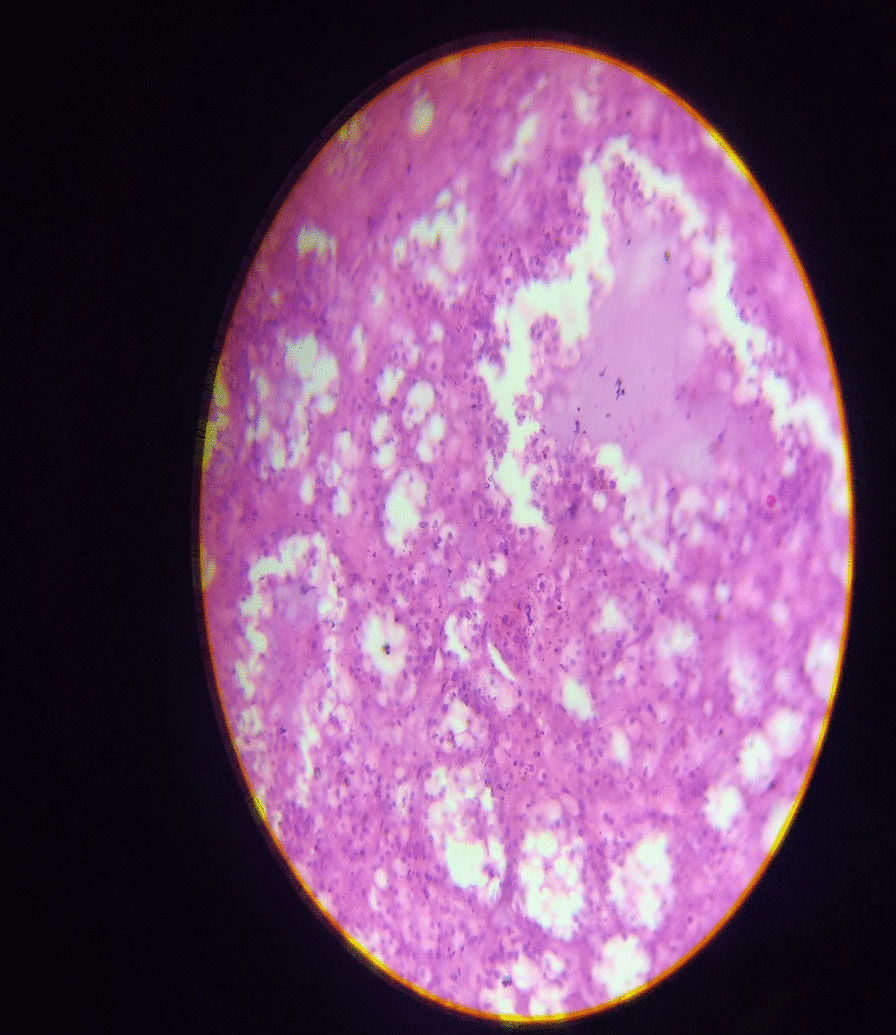
(histopathology revealed colloid adenomatous goiter with no evidence of malignancy)

Histopathology reported as colloid adenomatous goiter with no evidence of malignancy (Figs. 1, 2, 3, 4).

## Discussion

The thyroid gland is the first endocrine gland that develops during fetal embryology [3].

Failure of the thyroid to descend from the thyroid anlage region to its final location in front of the trachea is called ectopic thyroid. Ectopic thyroid can be present at any position from the foramen caecum to the base of the tongue to the mediastinum [2].

The most common ectopic thyroid location is the lingual thyroid. Excessive movement can lead to superior mediastinal or even paracardiac location [3].

Ectopic thyroids are common in females, with a female: male ratio of 4:1, and can occur at any age but are particularly observed during childhood and adolescence [7].

Ectopic thyroids are usually asymptomatic, but some local symptoms, such as dysphagia, dysphonia or upper airway obstruction, may be seen [4].

Radiological imaging studies such as CT scan and MRI may be helpful to determine the extent of the mass. A normally located thyroid gland with normal echogenicity, contour and size confirmed on ultrasound is highly likely to be functional, suggesting that an abnormal mass may be removed without the risk of postoperative hypothyroidism [4].

I 131 is not always observed in ectopic thyroid tissue; hence, scintigraphy is not always diagnostic [8].

Tissue biopsy can be performed using CT-guided fine needle aspiration or EBUS transbronchial needle aspiration [7]. It is especially important when malignancy is suspected.

Ectopic thyroid may become goitrous [5], and rarely benign or malignant neoplastic changes can occur in the ectopic tissue [6]. Nevertheless, these should be surgically resected due to the risk of malignant transformation, progressive enlargement, haemorrhage within causing respiratory obstruction and compression of neighbouring vital mediastinal organs [7].

Ectopic thyroid is one of the pathologies that should be considered when investigating cases of mediastinal tumors. The most common are lymphomas, germ cell tumors, substernal thyroid and neurogenic tumors [1].

Ectopic thyroid in the thorax without connection to the original gland in the neck is very rare, and only a few cases have been reported in the literature. It is important to differentiate between substernal goiter and ectopic goitre [1].

In our case, the patient was an elderly male with an incidentally detected mediastinal mass. Surgical excision was performed through sternotomy. Histopathology revealed a benign adenomatous goiter in the ectopic mediastinal mass with a normal (anatomically and functionally) orthotopic thyroid gland, which is a rare combination.

## Conclusion

Ectopic thyroid tissue is a rare cause of mediastinal masses; however, it must be considered as one of the differential diagnoses. Awareness of the possibility of benign or malignant transformation and life-threatening complications necessitates further investigation and surgical excision.

## Data Availability

Not applicable.
